# Omega 3 fatty acid inhibition of inflammatory cytokine-mediated Connexin43 regulation in the heart

**DOI:** 10.3389/fphys.2012.00272

**Published:** 2012-07-12

**Authors:** Jennifer R. Baum, Elena Dolmatova, Alex Tan, Heather S. Duffy

**Affiliations:** Beth Israel Deaconess Medical Center, Harvard Medical SchoolBoston, MA, USA

**Keywords:** arrhythmia, fibrosis, gap junction, inflammation, interleukin, myocardial infarction

## Abstract

**Background:** The proinflammatory cytokine Interleukin-1β (IL-1β), which increases in the heart post myocardial infarction (MI), has been shown to cause loss of Connexin43 (Cx43) function, an event known to underlie formation of the arrhythmogenic substrate. Omega 3 Fatty acids exhibit antiarrhythmic properties and impact IL-1β signaling. We hypothesize that Omega-3 fatty acids prevent arrhythmias in part, by inhibiting IL-1β signaling thus maintaining functional Cx43 channels. **Methods:** Rat neonatal myocytes or Madin-Darby Canine Kidney Epithelial (MDCK) cells grown in media in the absence (Ctr) or presence of 30 μM docosahexaenoic acid (DHA, an Omega-3 Fatty acid) were treated with 0.1 μM activated IL-1β. We determined Cx43 channel function using a dye spread assay. Western blot and immunostaining were used to examine Cx43 levels/localization and downstream effectors of IL-1β. In addition we used a murine model of MI for 24 h to determine the impact of an Omega-3 fatty acid enriched diet on Cx43 levels/localization post MI. **Results:** IL-1β significantly inhibited Cx43 function in Ctr cells (200.9 ± 17.7 μm [Ctr] vs. 112.8 ± 14.9 μm [0.1 uM IL-1β], *p*<0.05). However, DHA-treated cells remained highly coupled in the presence of IL-1β [167.9 ± 21.9 μm [DHA] vs. 164.4 ± 22.3 μm [DHA + 0.1 uM IL-1β], *p*<0.05, *n* = 4]. Additionally, western blot showed that IL-1β treatment caused a 38.5% downregulation of Cx43 [1.00 au [Ctr] vs. 0.615 au (0.1 μM IL-1β) which was completely abolished in DHA-treated cells (0.935 au [DHA] vs. 1.02 au [DHA + 0.1 μM IL-1β), *p* < 0.05, *n* = 3]. Examination of the downstream modulator of IL-1β, NFκβ showed that while hypoxia caused translocation of NFκβ to the nucleus, this was inhibited by DHA. Additionally we found that a diet enriched in Omega-3 Fatty acids inhibited lateralization of Cx43 in the post-MI murine heart as well as limited activation of fibroblasts which would lead to decreased fibrosis overall. **Conclusions:** Omega 3 Fatty acid treatment inhibited IL-1β-stimulated loss of Cx43 protein, and more importantly, inhibited loss of Cx43 function by inhibiting translocation of NFκβ. In the intact heart a diet enriched in Omega 3 Fatty Acids limited loss of Cx43 at the intercalated disk in the heart following MI. These data suggest that one of cardio-protective mechanisms by which Omega 3 Fatty acids work includes prevention of the pro-arrhythmic loss of Cx43 post MI and the attenuation of cardiac fibrosis after injury.

## Introduction

Gap junctions provide direct electrical continuity between myocytes in the functioning myocardium. These junctions are formed from connexin proteins which are four transmembrane domain proteins which oligomerize form a half channel known as a connexon. Connexons from apposing cells meet head-to-head across the extracellular space and form a full channel that allows for direct cytoplasmic continuity between the cells. In the heart this channel is a low resistance pore which allows for rapid electrical conduction through the working myocardium. Loss of gap junctions in cardiac injury is associated with increased arrhythmogenicity and Sudden Cardiac Death (Peters et al., 1997; Gutstein et al., 2001).

In 1978, Dyerberg et al. reported that the Greenland Inuit population had higher than average levels of the long chain *n-3* fatty acid (*n-3* LCFA now known as Omega 3 Fatty Acids or w3 fatty acids) and importantly they had an associated decreased prevalence of atherosclerotic disease (Dyerberg et al., 1978). This report set off decades of research into the mechanisms by which these LCFA might work and what the extent of their cardiovascular benefits might be. A recent meta analysis showed that dietary intake of w3 fatty acids is associated with a significant decrease in sudden cardiac death (35.1% decrease) (Musa-Veloso et al., 2011). While the understanding of the benefits of dietary intake of w3 fatty acids has come a long way, the molecular mechanisms by which these work are less understood. Although there have been several studies examining the molecular mechanisms by which w3 fatty acids alter cellular behavior it is still unclear the extent to which fatty acids directly alters cellular functions involved in cardiac electrophysiology and how they work to do so. Being as gap junctional conduction is a key player in normal cardiac conduction, in this study we examined the effects of w3 fatty acids on the levels, localization, and function of the primary ventricular gap junction protein Cx43.

The initial thought on how w3 fatty acids worked was that it intercalated itself into the cellular membranes thereby altering the fluidity of the membranes which then, in turn, altered ion channel behavior (Hallaq et al., 1990; Macleod et al., 1998; Leaf et al., 2002). More recent studies have shown that w3 fatty acids alter gene expression via activation of nuclear factor kappa beta (NFκβ) as well as direct interaction with transcription factors in the nucleus (Di Nunzio et al., 2009). Being as NFκβ alters the response of myocytes to the inflammatory cytokine Interleukin 1β, which is upregulated following myocardial infarction (MI) (Abbate et al., 2010), and we have shown that IL-1β affects the gap junction protein Connexin43 (Cx43) in both the nervous system (Duffy et al., 2000) in the injured heart (Baum et al., 2012), we hypothesized that one of the mechanisms for the anti-arrhythmic effects of a diet containing w3 fatty acids is by regulating Cx43 containing gap junctions following myocardial injury.

## Methods

### Mouse diets

All animal procedures were done with approval from the Animal Care Institute (IACUC) at Beth Israel Deaconess Medical Center and in compliance with NIH guidelines. C57 black mice were fed *ad libitum* diets either enriched for Omega 3 Fatty Acids (3% of total calories) or a matched diet of 3% lard (Harlan, Madison, WI) for 6 weeks starting from the age of 6 weeks. At the end of the diet period the mice from each diet group were split into control or MI groups. Both groups of mice were anesthetized and a small incision was made in the left thorax. Control mice were closed and allowed to recover for 24 h. MI mice had the left anterior descending coronary artery ligated at the place where the artery surfaced on the front of the heart. These mice were closed and allowed to recover for 24 h. After 24 h mice from all groups were sacrificed and hearts were excised for biochemical studies.

### Dye spread assay

Cell cultures-MDCK cells were grown in 35 mm dishes in a medium of DMEM (ATCC) supplemented with 10% fetal bovine serum (Sigma) and 1% penicillin-streptomycin (Cellgro) then incubated overnight in 1 μM interleukin-1β (Sigma) with or without 3 mM DHA. After incubation media was removed and cells were scraped with a razorblade and incubated for 5 min in 0.5% Rhodamine Dextran plus 2.5% Lucifer Yellow in 150 mM Lithium Chloride. Following PBS rinses (3 × 10 min) cells were fixed in 4% formaldehyde for 15 min then examined on a Leica 5500 inverted microscope. Images were taken from three areas (at center, and at 25% from both top, and bottom of image) of five separate dishes per treatment and dye spread was measured from the scrape line to the furthest edge of dye spread (Image J NIH Shareware). Statistical analysis was done using an ANOVA with Bonferroni correction.

### Cell culture

MDCK cells were plated either in 35 mm dishes or on glass cover slips for Western blot and Immunohistochemistry, respectively. Cells were maintained in DMEM + 10% Fetal bovine serum and 0.01% PenStrep until confluent. Cells were then treated with 0.01 mM IL-1β overnight then fixed in 4% formaldehyde and stained for Cx43 as described below.

### Western blot

Cell cultures and tissue samples were lysed in complete lysis buffer (50 mmol/L Tris-HCl pH 7.4, 0.25 mmol/L Na-deoxycholate, 150 mmol/L NaCl, 2mM EGTA, 0.1 mmol/L Na3VO4, 10 mmol/L NaF, 1 mmol/L PMSF, 1% Triton-X 100, ½tablet of Complete Protease Inhibitor (Roche Biochemicals, Indianapolis, IN). Lysates were sonicated for 30 s, maintained on ice for 30 min. then triturated and spun at 10,000 rpm for 10 min. Following removal of the pellet protein levels were tested using BCA protein Assay Kit (BioRad). Matched levels of total protein were mixed with loading buffer (2X laemini buffer + DTT), then run for Western blots using 10% SDS-PAGE gels. Gels were Commassie blue stained as loading control (Sohlenius et al., 1996) and then proteins were transferred to nitrocellulose membranes and probed for Cx43 (Sigma). Bands were analyzed by densitometry (Cx43/Commassie blue) using NIH Scion Image.

### Immunohistochemistry

Rapidly-frozen heart samples from fixed cell cultures or from epicardium of mouse hearts were sectioned (15 microns) using a Leica 3050S cryostat. Sections were fixed in 4% formaldehyde for 30 min at RT then incubated in 50 mM NH_4_Cl for 30 min to quench autofluorescence. Following quench, sections were blocked (PBS + 10% goat Serum + 0.4% Triton-X 100) for 1 h at RT then incubated with primary antibodies directed against Cx43 (Sigma) at 4°C overnight. Following 30 min rinse (3 × 10 min, PBS + 0.4% Triton-X 100) slices were incubated with secondary antibodies (Alexa Fluor, anti-mouse 488 and anti-rabbit 595) for 1 h at RT. Slices were rinsed for 50 min (5 × 10 min), and mounted on glass microscope slides with Vectashield anti-fade agent (Vector Laboratories, Burlingame, CA) and examined using a Zeiss Axiophott 200 M equipped with both FITC and Texas Red filters.

Epicardial mapping-Mice (*n* = 3 per group) were anesthetized with 5% vaporized isoflurane and maintained at 2–3% vaporized flow. The chest was opened and an electrode array (10 × 6 mm) was placed on the left ventricle and electrical signals were collected using a UnEmap system (Aukland, New Zealand). This system amplifies, records, stores and analyzes time of occurrence of electrical signals, and graphically displays the data as activation maps. Rates of inducibility into VT were compared by ANOVA and considered to be significantly different at *p* < 0.05.

## Results

### Omega 3 fatty acids limit IL-1 beta-induced loss of gap junction function

**Figure 1:**
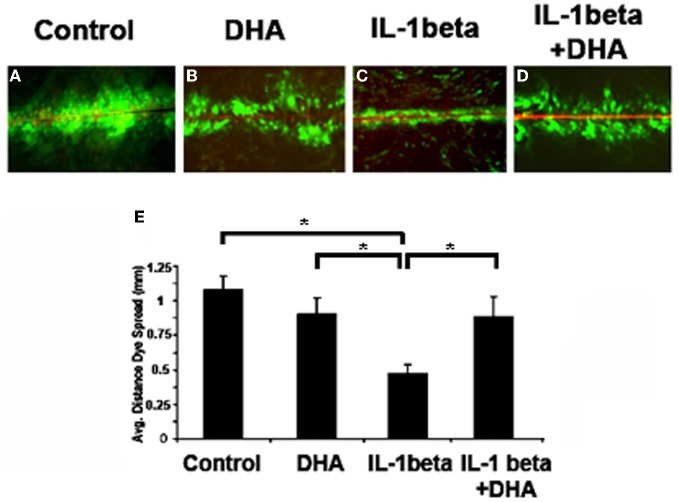
**Dye spread in MDCK cells.** Lucifer Yellow (LY, green) easily passed through gap junctions in control cells **(A)**, an event not altered by DHA treatment alone **(B)**. In contrast IL-1β treatment caused a significant decrease in coupling **(C)** which was inhibited in the presence of DHA **(D)**. Quantification of dye spread is seen in **E**. Texas Red Dextran (red) was used to mark broken cells. Cells with red in them were not included in the analysis. *N* = 3, *p* < 0.05.

To determine the effects of w3 fatty acids on gap junction function we used a standard scrape-loading method to examine Cx43 channel function (Figure 1). Cells were incubated with IL-1β or IL-1β plus DHA and the distance that Lucifer Yellow dye spread was measured and used as an indicator of gap junction function. Control cells passed Lucifer Yellow (LY) though gap junctions more than 1.0 mm on average (Figure 1A). Incubation of control cells with DHA alone had no significant effect on LY transfer. In contrast, incubation of cells with IL-1β significantly decreased dye spread to 0.48 mm showing a loss of gap junctional function. When cells were pretreated with DHA then incubated with IL-1β, channel function was maintained at levels comparable to normal despite the presence of IL-1β. These data indicate that w3 fatty acids inhibit the IL-1β-induced loss of Cx43 function.

### Effects of DHA on Cx43 levels

**Figure 2:**
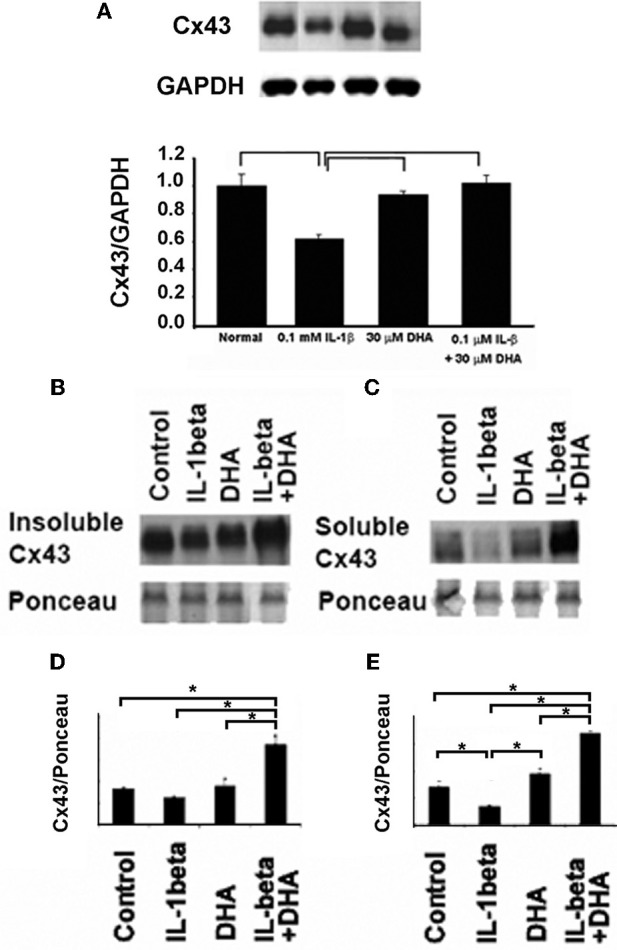
**Western-blotting results for the effect of DHA treatment on changes in total (A) membrane (B), and cytoplasmic (C) Cx43 in neonatal rat ventricular myocytes (NRVM).** IL-1β application led to a significant loss of total Cx43 **(A)**. This loss was seen in both soluble and insoluble fractions (**B** and **C**). DHA inhibited this effect. Quantification of the insoluble **(D)** and soluble **(E)** Cx43 fractions is shown below. Ponceau staining was used as a loading control. Densitometry of the bands (*n*=3) is shown *p*<0.05.

To assess whether DHA changed the total levels of Cx43 in cardiac myocytes, either at the cell membrane or within the cytoplasm of the cell, we performed Western-blotting of total, soluble (cytoplasmic) and insoluble (membrane) fractions of neonatal rat ventricular myocytes (Figure 2). Examination of total levels of Cx43 showed that IL-1β decreased Cx43. DHA treatment alone had no effect on control cells but inhibited the loss of Cx43 in the presence of IL-1β (Figure 2A). To determine if DHA was affecting the membrane localized Cx43 preferentially, which would suggest that the mechanism of increased Cx43 was via membrane stabilization, we examined Cx43 levels in membrane (Figure 2B, Insoluble Cx43) vs. cytosolic (Figure 2C, Soluble Cx43) fractions of control, DHA alone, IL-1β and IL-β + DHA treated cardiac myocytes. We found that IL-1β did not significantly decrease the amount of Cx43 found in the insoluble membrane fraction of the cells. Instead the decrease in Cx43 was seen only in the cytosolic fraction suggesting that IL-1β induced loss of Cx43 is due to changes in the production of Cx43 rather than in its stabilization at the cell membrane. Pretreatment with DHA significantly increased both the insoluble membrane form of Cx43 showing that it has some affects on stabilization of Cx43 within cell membranes but in addition it also increased the soluble cytosolic form of Cx43 suggesting it was also impacting gene expression and/or protein degradation of Cx43. These data indicate that the maintenance of gap junction function seen with DHA treatment may be via two pathways, one being increased open channels in the cellular membranes and the second being from increased expression of Cx43.

### NFκβ translocation is inhibited by DHA

**Figure 3:**
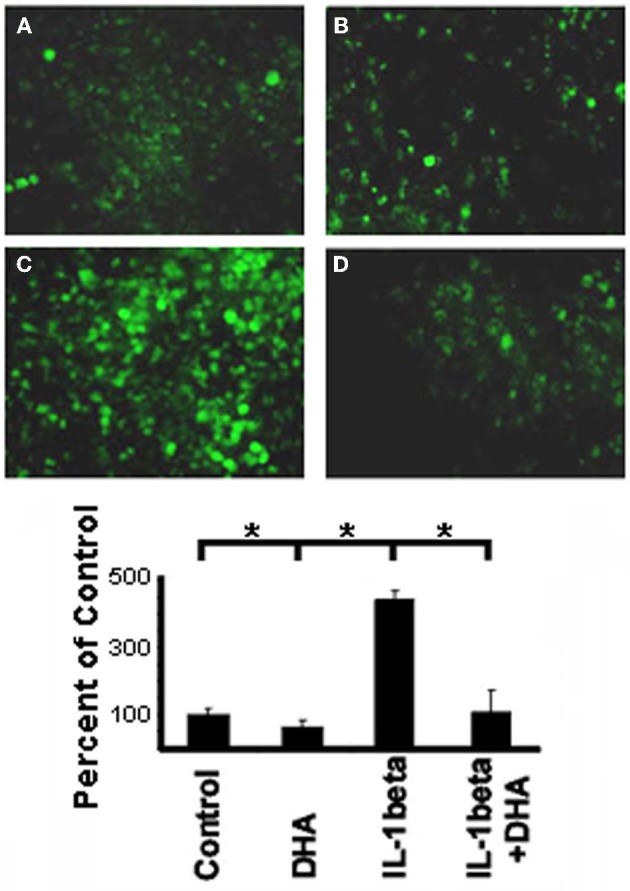
**Immunofluorescence of activated p65 NFκB in NRVM.** Control cells show low levels of NFκB staining **(A)** which is not change with DHA incubation alone **(B)**. In contrast, stimulation with IL-1β led to significant increases in NFκB activation **(C)**. DHA incubation inhibited activation of NFκB back to control levels **(D)**. (*n*=3, *p*<0.05).

NFκβ is a downstream mediator of IL-1β signaling which has been shown to bind to the Cx43 promotor and regulate Cx43 levels (Alonso et al., 2010). Therefore, to determine if the mechanism by which DHA inhibits IL-1β-induced loss of Cx43 was via this canonical IL-1β signaling pathway we examined the effect of DHA on NFκβ activation by immunostaining for the active p65 form of NFκβ (Figure 3) in NRVMs. Control cells showed low levels of p65 NFκβ (Figure 3A) but IL-1β treatment of NRVM caused a significant increase in active p65 NFκβ (Figure 3B, *p* < 0.05). DHA alone had no effect on p65 NFκβ levels (Figure 3C). In contrast to the IL-1β treated cells, cells incubated with DHA and IL-1β had p65 NFκβ levels that were not significantly different from control cells (Figure 3D). This suggests that one mechanism of action of w3 fatty acids may be by blocking p65 NFκβ activation thereby inhibiting the IL-1β signaling cascade (Figure 3).

### Effects of DHA *in vivo*

**Figure 4:**
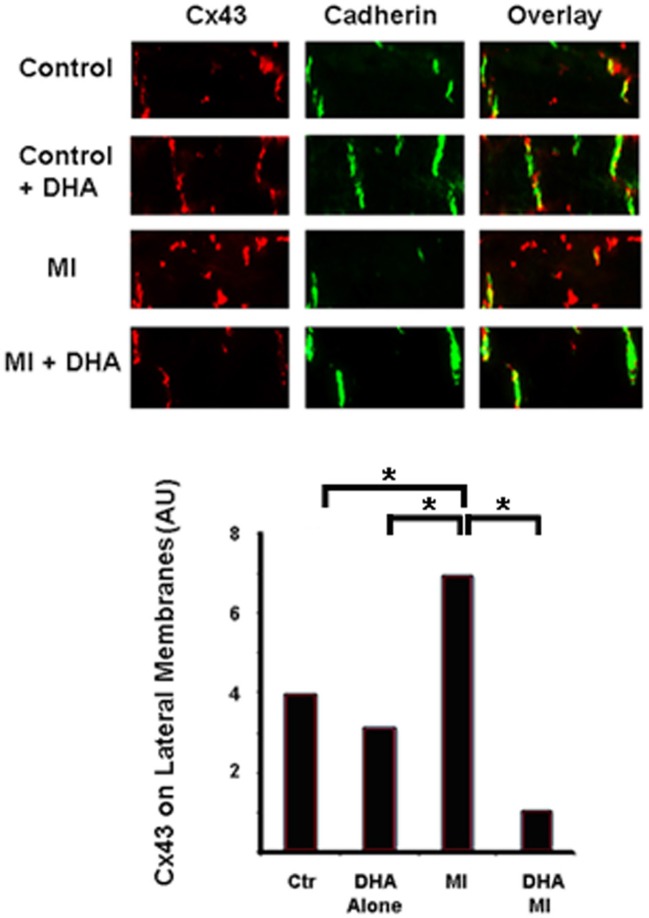
**Immunostaining of Cx43 distribution in control and DHA-treated mice following myocardial infarction.** Cx43 (red) is found at the intercalated disks (Cadherin was used as a marker of the disk, green) in control animals in both diet groups (Control and Control + DHA). As expected MI caused lateralization of Cx43 (MI). This lateralization was completely inhibited in animals fed the DHA enriched diet (MI + DHA). *n* = 3, *p* < 0.05.

Our previous experiments showed that DHA normalizes Cx43 under inflammatory conditions in cell culture. To determine the effects of DHA *in vivo* we pretreated mice with two different diets for six weeks. The first group of mice received a DHA enriched diet (2.5%) while control mice were fed a matched fat diet (2.5% lard/corn oil). We then ligated the left anterior descending coronary artery (LAD), waited for 24 h and then harvested the hearts. Figure 4 shows the results of immunostaining from control and DHA pretreated mouse hearts. Cx43 can be seen in red, while Cadherin (used to mark intercalated disks) is in green. Increased lateralization of Cx43 can be seen in control mice following coronary occlusion (CO). However, DHA pre-treatment seemed to completely reverse this lateralization. This suggests that DHA pretreatment may be able to prevent Cx43 displacement and ensure the continued maintenance of normal gap junction localization following MI.

### Impact of DHA on fibroblast to myofibroblast transformation

**Figure 5:**
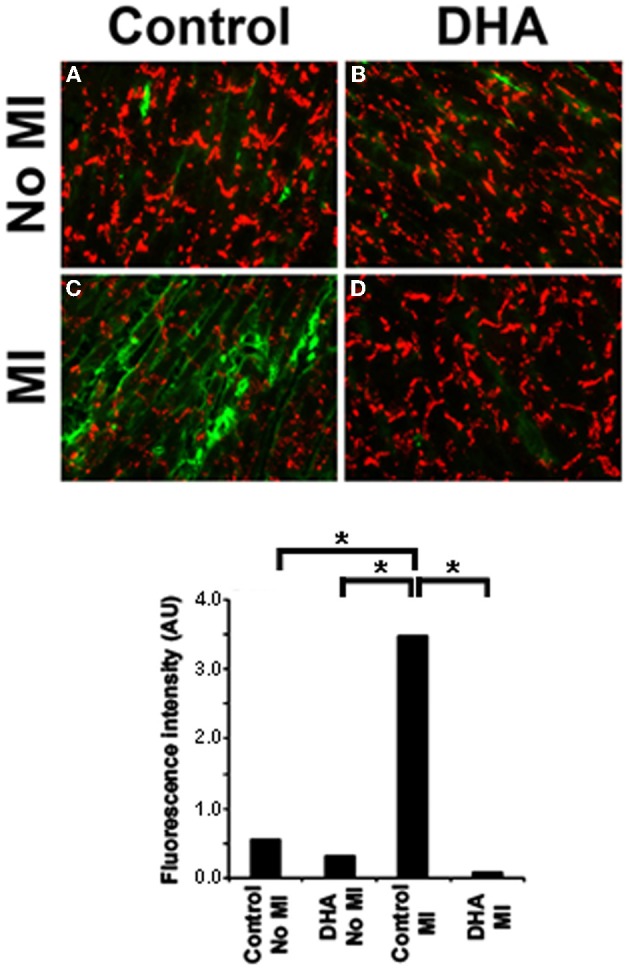
**Immunostaining for Cx43 (red) and SMA (green) in mice following coronary occlusion (CO) that received either a DHA or matched fat diet (Control).** Control animals showed only very low levels of Smooth Muscle Actin staining regardless of their diet (**A** and **B**). In MI animals without DHA the number of SMA positive cells significantly increased after CO (**C**, MI). DHA pretreatment caused SMA positive staining to be restricted to vessels **(D)**. Lower panel shows the quantification of SMA staining intensity. *n*=3, *p*<0.05.

Our previous work has shown that fibroblast to myofibroblast transformation following CO greatly affects normal Cx43 distribution in cardiac myocytes (Baum et al., 2012). We therefore decided to examine whether or not DHA could prevent myofibroblast activation *in vivo* and thereby maintain normal Cx43 localization post-MI. To answer this, we used our mouse diet model and then sectioned and immunostained the hearts for the presence of Smooth Muscle Actin, a marker of myofibroblasts (activated fibroblasts). Figure 5 shows that normal mouse hearts (Figure 5A) or hearts from mice that ate the DHA enriched diet but did not have their coronary artery ligated (Figure 5B) have little or no myofibroblasts but that MI induces the activation of fibroblasts in the injured region (Figure 5C). In contrast, mice fed a DHA enriched diet showed a significant decrease in SMA staining following MI as compared with control or DHA alone mice (Figure 5D). Quantification of the SMA staining in heart sections is shown in Figure 5E. This data show that DHA inhibits fibroblast to myofibroblast transformation post MI. Further studies to examine the overall levels of fibrosis in these hearts are needed to determine if blockade of fibroblast transformation could help decrease fibrosis in the injured heart.

### Effect of a DHA enriched diet on arrhythmia inducibility in the intact heart

**Figure 6:**
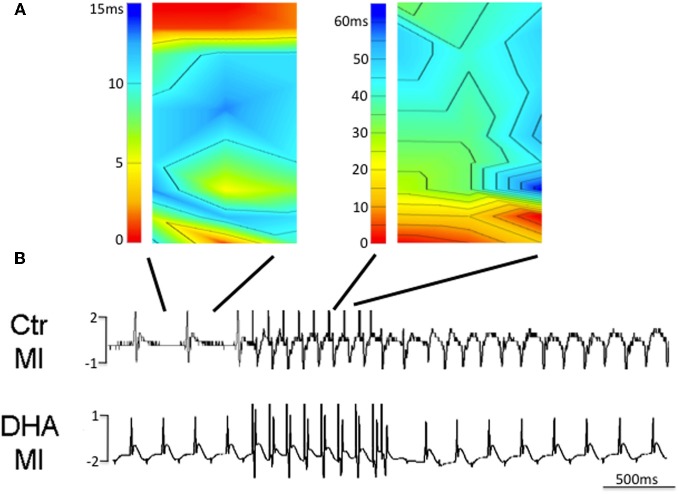
**Isochronal maps of sinus rhythm (SR) and induced ventricular tachycardia (VT) with a single premature stimulus (120 ms drive cycle, S2 60 ms) in a mouse fed a normal diet after MI (A and B upper trace, *n* = 3).** In contrast, mice fed diets enriched in DHA were unable to be induced to VT with the same prematurity (**B**, lower trace, *n* = 3).

To determine if chronic ingestion of a diet rich in w3 fatty acids was able to limit arrhythmogenicity in a model of MI animals from all groups were subjected to epicardial mapping for ventricular tachycardial (VT) inducibility. We found that control animals were unable to be stimulated into VT but while animals which ate a normal diet had normal sinus rhythm, they were easily induced into VT (Figure 6A and upper trace in **B**). In contrast, mice fed a diet enriched in w3 fatty acids were unable to be stimulated into VT (Figure 6B, lower trace). These data suggest that a diet enriched in DHA but eaten prior to any cardiac injury may have antiarrhythmic effects following MI.

## Discussion

Inflammatory processes in the heart lead to loss of Cx43 thus therapeutically targeting inflammation may decrease this loss and limit formation of arrhythmias. Studies have shown that diets enriched in w3 fatty acids lead to an attenuated inflammatory response by limiting the activation of the IL-1β receptor and limiting IL-1β cellular signaling through its nuclear mediator, NFkB (Adkins and Kelley, 2010). In this study we examined the changes in NFkB following IL-1β stimulation with and without DHA, the w3 fatty acids and found that w3 fatty acids decreased NFkB translocation to the nucleus. Additionally, the loss of cellular coupling seen with IL-1β treatment was abrogated in the presence of DHA. Additionally total levels of Cx43 in the cellular membranes was increased in the presence of DHA which is likely due to stabilization of Cx within the membrane by the intercalation of the into the cellular membrane (Adkins and Kelley, 2010). Thus, DHA appears to affect coupling at two levels. First, DHA treatment appears to limit IL-1β signaling which has been shown to decrease Cx43 levels and function. Additionally DHA increased Cx43 within cellular membranes, helping to maintain coupling. Maintenance of cellular coupling throughout the ventricle is a requirement for normal electrical conduction through the heart. Studies have shown that heterogeneous loss of coupling increases ventricular arrhythmias by promoting regions of slowed conduction intermixed with regions of normal conduction. These regions of slowed conduction lead to formation of reverse conduction as electrical propagation from normal areas enters non-repolarizing regions ahead of the slow wave front. The figure of eight re-entrant circuits that are set up by this abnormal conduction pattern can then anchor spiral waves and lead ventricular tachycardia and fibrillation. We found mice fed high DHA diets were not inducible into VT under any circumstance. Thus, the changes we found in Cx43 and myofibroblast activation appeared to be at least part of a stabilizing substrate in these animals thus limiting the arrhythmogenicity of their hearts. It is of interest to note that these studies show that a diet given prior to the cardiac injury is capable of preventing subsequent damage after cardiac injury suggesting that the mechanism of action is to stabilize the healthy myocardium rather than to reverse remodel the injured myocardium.

One of the hallmarks of myocardial injury is the presence of Cx43 on the lateral membranes of cardiac myocytes as opposed to their normal localization at the intercalated disk (Peters et al., 1997). The initial thought was that these channels allowed for transverse conduction leading to slowing of conduction in the longitudinal direction and subsequent reentrant arrhythmias. Subsequent studies showed that the channels on the lateral membranes were non-functional (Yao et al., 2003) and that the anisotropic ratio, which would be expected to decrease if transverse conduction were increased, actually was larger in injured myocardium. Attempts have been made to identify the mechanisms by which lateralization occurs (Kieken et al., 2009) as well as identify therapies which limit loss of gap junctional coupling (Kjolbye et al., 2008; Wiegerinck et al., 2009). To date, therapies which decrease lateralization have not been found (Macia et al., 2011). In this study we have identified not a therapy *per se*, but a method by which lateralization of Cx43 can be limited following MI. We found that mice who had a prior exposure to a diet enriched in w3 fatty acids limited lateralization of Cx43 following MI. This decrease in lateralization was associated with maintenance of Cx43 at the intercalated disk so the w3 fatty acid was not just altering internalization of Cx43. In addition, examination of smooth muscle actin levels in the w3 fatty acids fed mice showed a marked decrease in the level of fibroblast activation. Based on our data showing w3 fatty acids decrease IL-1β signaling through NFκβ we hypothesize that the diet decreased the inflammatory processes in the heart thus limiting fibroblast activation.

In addition, following MI or other heart injury inflammatory processes begin the healing process which, when uncontrolled, leads to fibrosis which also contributes to formation of the arrhythmogenic substrate. Immune cells such as monocytes and macrophages migrate to the site of injury where they release pro-inflammatory cytokines. These cytokines affect a wide range of cellular processes and contribute to the transformation of resident fibroblasts to the activated myofibroblast phenotype. It is these myofibroblasts which then, through secretion of extracellular matrix components and regulators, lay down the collagenous bundles seen in fibrosis. In addition, during active healing these cells continue to produce such cytokines as TGF-β, IL-6, and important for this discussion, IL-1β. Our previous studies have shown that IL-1β causes a decrease in Cx43 based cellular coupling in both the nervous system (Duffy et al., 2000) and the heart (Baum et al., 2012). In the heart the presence of myofibroblasts in injured regions leads to heterogeneous loss of Cx43 and formation of an arrhythmogenic substrate. The present studies show that a diet enriched in w3 fatty acids may limit activation of fibroblasts suggesting that it may decrease post-MI fibrosis. This decrease, combined with maintenance of functional gap junctions in the injured heart may be why w3 fatty acids may exhibit anti-arrhythmic characteristics.

In conclusion, our studies show that a regular diet which contains w3 fatty acids prior to any cardiac injury limit the loss of Cx43 induced by IL-β signaling through NFκβ. This protection from the loss of coupling is likely to cause a decrease in the arrhythmogenic potential of the heart. This could explain why studies have shown that almost half the reduction of risk for people on w3 fatty acids supplementation was due to a decrease in arrhythmic events and Sudden Cardiac Death (Levantesi et al., 2010). These data suggest that early and consistent dietary supplementation with w3 fatty acids may limit cardiovascular risk overall when part of the normal diet prior to any cardiac injury.

### Conflict of interest statement

The authors declare that the research was conducted in the absence of any commercial or financial relationships that could be construed as a potential conflict of interest.
